# Communicating effectively with patients about vaccination: A systematic review of randomized controlled trials

**DOI:** 10.14745/ccdr.v49i78a05

**Published:** 2023-08-01

**Authors:** Chloé Desjardins, Manon Denis-LeBlanc, Christine Paquette Cannalonga, Malek Rahmani, Teresa A Gawargy, Pierre-Marc Dion, Jennifer Lacroix Harasym, Salomon Fotsing, Maria Cherba, Nigèle Langlois, Sylvain Boet

**Affiliations:** 1 Francophone Affairs, Faculty of Medicine, University of Ottawa, Ottawa, ON; 2 Department of Family Medicine, University of Ottawa, Ottawa, ON; 3 Institut du Savoir Montfort, Ottawa, ON; 4 Department of Communication, Faculty of Arts, University of Ottawa, Ottawa, ON; 5 Health Sciences Library, University of Ottawa, ON; 6 Faculty of Education, University of Ottawa, Ottawa, ON; 7 Departments of Anesthesiology and Pain Medicine, University of Ottawa, Ottawa, ON; 8 Department of Innovation in Medical Education, University of Ottawa, Ottawa, ON; 9 Clinical Epidemiology Program, Ottawa Hospital Research Institute, Ottawa, ON; 10 Keenan Research Centre, Li Ka Shing Knowledge Institute, Toronto, ON

**Keywords:** communication, randomized controlled trials, vaccines, vaccine hesitancy

## Abstract

**Background:**

Good communication between healthcare professionals and their patients is essential to enlighten the benefits and risks of vaccination. Despite the availability of effective vaccines, reluctance prevails, sometimes fuelled by sub-optimal communication leading to a lack of trust. An evaluation of the effectiveness of a communication strategy for which healthcare professionals are trained has yet to be carried out.

**Objective:**

Systematic review of studies with a randomized controlled trial (RCT) to define and evaluate the impact of healthcare professionals’ communication on patients’ vaccine adherence.

**Methods:**

We performed a structured search on Medline, Embase, CENTRAL, PsycINFO and CINAHL. The studies selected include those involving healthcare professionals authorized to administer vaccines according to Canadian guidelines. Primary outcomes include vaccination rate or vaccine hesitancy rate.

**Results:**

Nine articles were included. Five studies (n=5) reported intervention effectiveness according to vaccine adherence. The results are largely represented by parental vaccine hesitancy for human papillomavirus (HPV) or childhood vaccination, while three studies (n=3) target the general population. The risk of bias relative to the studies is either low (n=7) or of some concern (n=2).

**Conclusion:**

The effectiveness of communication varies according to the studies and knowledge acquired through training. Future studies will need to examine communication with healthcare professionals in order to establish a consensus on optimal and appropriate training.

## Introduction

Vaccination is effective in preventing many diseases and their serious forms. However, some patients are reluctant to be vaccinated, despite the potentially harmful consequences for their health and that of the population as a whole. This hesitancy stems from multiple, complex and sometimes interconnected factors ((1–7)). Possible reasons include a lack of trust in healthcare professionals and institutions, healthcare professionals’ lack of patient communication skills ((4,5,7)), or difficulties in navigating the sometimes contradictory information available ((1–3,5)).

Physician-patient communication is defined in the literature as a key component of the therapeutic relationship, enabling the development of a bond of trust that leads to optimal care ((5,7–9)). The bond of trust is important when discussing vaccination, since the decision-making process has an impact on individual and community safety ((1)). Given the importance of communication in healthcare decision-making, it is possible that a communication intervention with healthcare professionals could influence vaccine adherence. Given the coronavirus disease 2019 (COVID-19) pandemic and its repercussions, including the lack of educational resources in patient communication skills, a communication intervention is all the more important to address the limitations of healthcare institutions and mistrust of the COVID-19 vaccine. In the absence of intervention, current limitations may lead to mistrust of future vaccines in times of health crisis. The effectiveness of intervention has yet to be systematically evaluated.

### Objectives

We conducted a systematic review of randomized controlled trials (RCTs) to define and evaluate the impact of healthcare professionals’ communication on patients’ vaccine adherence.

## Methods

### Protocol and registration

This systematic review was conducted in accordance with AMSTAR 2 (A Measurement Tool to Assess Systematic Reviews) standards ((10)) and the Preferred Reporting Items for Systematic Reviews and Meta-Analyses (PRISMA) guidelines ((11)). The protocol has been registered with the International Prospective Register of Systematic Reviews (PROSPERO) (CRD42022330645).

### Eligibility criteria

All RCTs in which participants were healthcare professionals authorized to administer vaccines (doctors, nurses, pharmacists and resident physicians) were eligible. We included studies in which communication on vaccine adherence was the main intervention. We excluded studies in which the healthcare professionals were medical, nursing or pharmaceutical students (not authorized to administer vaccines according to Canadian guidelines). We also excluded studies where the intervention was aimed at patients rather than healthcare professionals. Non-peer-reviewed articles, conference abstracts, letters, editorials and commentaries were not eligible.

### Information sources

Two electronic search reviews ((12) were carried out, a Medline search strategy and a translation of the CINAHL RCT Filter search. MEDLINE^®^ ALL via Ovid, Embase Classic + Embase via Ovid, Cochrane Central Register of Control Trials via Ovid, APA PsycINFO via Ovid and CINAHL via EBSCO were consulted.

### Search

The search strategy (**Supplemental material A**) was developed by an information specialist with the research team and revised by a second information specialist as suggested in the Peer Review of Electronic Search Strategies (PRESS) guide ((12)). Eligibility criteria (**Box 1**) included no language or publication date limits. A filter for published RCTs was applied ((13)). The search strategy was developed in Medline and then translated into the other databases. Key search concepts included MeSH terms related to vaccine adherence, healthcare professionals and communication. Only studies published and available in French or English were considered. The list of references cited in the included studies was also searched. The final list of included studies was reviewed by content experts to confirm their relevance.

### Box 1: Search strategy eligibility criteria

**Population:** healthcare professionals authorized to administer vaccines (physicians, nurses, pharmacists and resident physicians)

**Intervention:** communication training for healthcare professionals to be used during vaccination consultations only

**Comparison:** a control group of healthcare professionals who received no communication intervention

**Outcome:** vaccine adherence, defined as receiving, intending to receive or being less reluctant to receive the series of disease-preventing vaccines according to the schedule suggested by the national immunization authority

**Study date:** no limit

**Method: **randomized controlled trial

**Publication language:** no initial limit

**Publication date:** no limit

### Selection of studies

Studies were uploaded to a web-based software program, Covidence (version 2.0, Veritas Health Innovation, Melbourne, Australia) (14), and duplicates were removed. A pilot assessment tool, developed by the research team and tested on 30 randomly selected articles (**Supplemental material B**), was refined until subjectively acceptable agreement was established among the judges. Evaluation of each level of inclusion was carried out by pairs of independent reviewers, and conflicts were resolved by a third party.

### Data extraction

A data extraction grid (**Supplemental material C**), developed by the research team, was tested by the same reviewers. Extraction was performed in duplicate by pairs of reviewers and consensus by a third party. Extracted data include publication characteristics (name of lead author, year of publication, data collection sites), study characteristics (objective, study design and context, number of healthcare professionals, outcomes), type of healthcare professional, intervention details and results.

### Risk of bias inherent in each study

Pairs of reviewers assessed included studies for risk of bias according to the Risk of Bias Tool 2 for Randomized Controlled Trials (RoB 2) (15). The tool assesses the risk of bias attributed to study design, conduct and data reporting. For each area, a questionnaire is used to establish the level of risk as “low,” “some concern” or “high.” All areas must be predominantly low risk for the study to be considered reliable ((15)).

### Data summary

A description of all included studies is presented in tables containing information on demographic, clinical and methodological quality. The results are summarized qualitatively, given the heterogeneity of the included studies.

## Results

### Selection of studies

**Figure 1:**
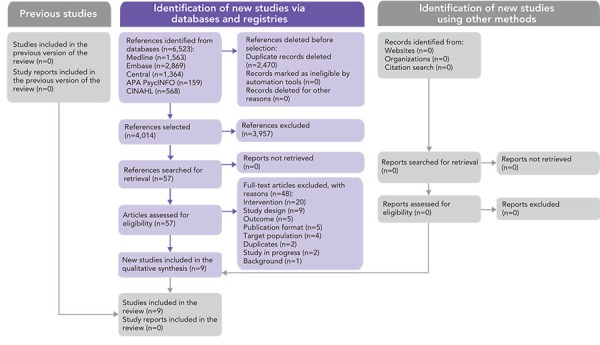
PRISMA 2020^a^ Flow Chart Abbreviation: PRISMA, Preferred Reporting Items for Systematic Reviews and Meta-Analyses ^a^ Page *et al.* ((11))

The search identified 6,484 studies. After eliminating duplicates, 4,014 studies were assessed for eligibility, including 57 full-text articles, 48 excluded studies and 9 included studies (**Figure 1**).

### Characteristics of selected studies

The included studies (n=9) employed communication training in a variety of formats targeting different knowledge areas, including understanding the virus, how the vaccine works, assertive communication, effective recommendations and the patient perspective. The vaccination context was childhood diseases (n=2), pneumonia/influenza (n=3), or human papillomavirus (HPV) (n=4). Six studies ((16–21)) focused on parental vaccine hesitancy, and three on adult vaccine hesitancy ((22–24)). General characteristics are shown in **Table 1**.

**Table 1:** Key features of included studies

First author, year	Country of data collection	Type of study	Background	Sample size (n), Age/sex (%)	Population	Study duration and format	Study objective(s)	Risk of bias
Abdel-Qader, 2022 ((22))	Jordan	RCT	Private practice of pharmacists and physicians	320 practitioners Age: NR Gender: 56 F vs. 43 M (intervention); 55 F vs. 45 M (control)	Doctors; pharmacists	16 online training sessions	To study vaccine hesitancy and evaluate the effectiveness of a collaborative physician-pharmacist intervention to improve adult COVID-19 vaccine hesitancy.	Some concern
Boom, 2010 ((16))	United States	RCT	Community practices in paediatric and family medicine	189 practitioners Age: NR Gender: NR	Doctors	One year; training one hour/day during lunch break	To evaluate the effectiveness of a university-based continuing education intervention aimed at increasing childhood vaccination rates in paediatric and family medicine practices in a large metropolitan area.	Low risk
Brewer, 2017 ((17))	United States	RCT	Paediatric and family medicine clinics	30 clinics (number of practitioners NR) Age: NR Gender: NR	Doctors; nurses; unspecified (i.e. health professionals or authorized personnel)	Four one-hour clinical training sessions	To determine the effectiveness of training providers to improve their recommendations using presumptive announcements or participatory conversations for HPV vaccine coverage.	Low risk
Dempsey, 2018 ((18)	United States	RCT	Primary care practices	16 clinics/188 practitioners Age: NR Gender: NR	Doctors; nurses; unspecified (i.e. health professionals or authorized personnel)	Series of two training sessions at team development meetings over six months	To evaluate the effect of a 5-component HPV vaccine communication intervention conducted by healthcare professionals on adolescent HPV vaccination.	Low risk
Gatwood, 2021 ((23)	United States	RCT	Two regional community pharmacy chains	96 pharmacies (number of practitioners NR) Age: NR Gender: NR	Pharmacists	Duration of training not reported; results were counted for a period of six months pre-intervention and post-intervention	To evaluate the impact of a communication training program to improve pharmacist promotion of pneumococcal vaccine among high-risk adults in Tennessee. The aim was to make it easier for pharmacists to address each patient’s beliefs and attitudes toward vaccination, particularly adults with chronic illnesses that put them at high risk of invasive pneumococcal infection.	Low risk
Gilkey, 2019 ((19))	United States	RCT	Cook Children's outpatient clinics	25 clinics/77 practitioners Age: NR Gender: NR	Doctors	One hour of clinical training	To evaluate the efforts of a paediatric health system to improve HPV vaccination coverage among adolescent patients. The objectives were to assess the extent to which a quality improvement (QI) program reached clinics and physicians, and the program’s impact on HPV vaccination coverage.	Low risk
Henrikson, 2015 ((20)	United States	RCT	Outpatient paediatric and family medicine clinics	56 clinics/526 practitioners Age: NR Gender: 68 F vs. 32 M (intervention); 64 F vs. 36 M (control)	Doctors	45-minute training session; 10-month intervention	To test whether a new communication intervention targeting physicians can improve physician confidence in communication and reduce vaccine hesitancy among mothers of infants.	Some concern
Muñoz-Miralles, 2021 ((24)	Spain	RCT	Urban and rural primary healthcare centres	57 practitioners Age: NR Gender: NR	Doctors; nurses	Duration of training not reported; one-year intervention	To determine the effectiveness of a brief intervention to increase influenza vaccination coverage compared with the usual advice in people who refuse it, and to record the main reasons for refusing to be vaccinated.	Low risk
Szilagyi, 2021 ((21))	United States	RCT	Paediatric primary care practices	48 clinics/234 practitioners Age: NR Gender: NR	Doctors	Three 20–30 minute online training modules; 6-month intervention	To evaluate the effect of online communication training for clinicians on missed HPV vaccination opportunities overall and during healthcare visits, acute and chronic illness visits, and on adolescent HPV vaccination rates.	Low risk

Abbreviations: COVID-19, coronavirus disease 2019; F, female; HPV, human papillomavirus; M, male; NR, not reported; RCT, randomized controlled trial

### Summary of results

Among the studies (n=9) included, the effectiveness of the interventions varied greatly according to the training format (5 effective ((17,18,21,22,24)); 3 no significant difference ((16,19,20)); 1 ineffective ((23))). A descriptive analysis of the communication adopted and its results are presented below. The measurement tools, primary outcomes and results with statistical significance are summarized in **Table 2**.

**Table 2:** Detailed results of included studies

First author, year	Results measurement tool(s)	Name of primary outcome(s)	Conclusion of primary results
Abdel-Qader, 2022 ((22))	Pre and post-intervention self-report survey assessing vaccine hesitancy and resistance from a physician’s perspective. Pre and post-intervention self-report survey assessing vaccination status. Pre and post-intervention self-report survey assessing knowledge, attitude and beliefs about COVID-19 vaccines.	The impact of collaborative physician-pharmacist training on COVID-19 vaccine hesitancy and resistance. Proportion of patients vaccinated before and after intervention.	The proportions of COVID-19 vaccine hesitancy and resistance were significantly reduced (20.1% and 7.8% vs. 64.3% and 35.7%, *p*<0.05), including one month after training (3.3% vs. 11.1%). The proportion of subjects vaccinated increased considerably (51.6% vs. 0.0%) one month after training. There was no significant difference in the proportion of patients vaccinated between the intervention and control groups.
Boom, 2010 ((16))	The Clinical Assessment Software Application (CASA) produced by the CDC (data entry and vaccination database).	Immunization rate for children aged 12 to 23 months.	There was no significant difference in the mean percentage of up-to-date vaccination for the control and intervention groups (19–23 months) (44% vs. 51%, *p*<0.05). After one year, there was a significant difference between the mean percentages of up-to-date vaccination for the control practices (41%) and the intervention practices (52%, *p*<0.05).
Brewer, 2017 ((17))	Data on vaccine coverage, specialty, number of patients, patient gender and patient eligibility for state-funded vaccines according to the North Carolina Immunization Registry (NCIR).	HPV vaccination rate in patients aged 11 to 17 years.	Presumptive announcement training showed a significant increase in HPV vaccination initiation at 6 months in 11 and 12-year-old adolescents vs. the control group (5.4% difference, 95% CI: 1.1%–9.7%). There was no significant difference for the conversation training. There was no significant difference in the 13 to 17-year-olds in the two groups.
Dempsey, 2018 ((18)	Vaccination data were extracted from each practice’s electronic medical record. To ensure completeness, this data was supplemented by data from the Colorado Immunization Information System.	HPV vaccine series initiation (one dose).	HPV vaccine initiation was significantly higher in adolescents in intervention practices (aOR: 1.46; 95% CI: 1.31–1.62) as was vaccine dose completion (aOR: 1.56; 95% CI: 1.27–1.92) compared to the control groups.
Gatwood, 2021 ((23)	Vaccine distribution records (pneumococcus, influenza, herpes zoster) provided by Walgreens in the Memphis and Nashville, Tennessee areas. Community vaccination beliefs and behaviours were compiled through an online survey facilitated by QuestionPro (Austin, Texas).	Increase in the rate of pneumococcal vaccination.	Compared to the Nashville area, people in the Memphis area showed less agreement that vaccines are a good way to protect against disease (73.8% vs. 79.7%, *p*<0.05), indicating a lower likelihood of following vaccine recommendations (73.4% vs. 78.3%, *p*<0.05) and more concern about side effects (47.1% vs. 35.8%, *p*<0.0001). Between the 6-month periods in 2018 and 2019, pneumococcal vaccine rates administered (on all patients) decreased in both regions.
Gilkey, 2019 ((19))	EMR to assess vaccine coverage. Vaccination in patients aged 12 to 14 years using standardized EMR queries.	HPV coverage (minimum one dose) for: 1) Model 1 (an intention-to-treat analysis of all doctors randomly assigned to the intervention and control groups); 2) Model 2 (a sensitivity analysis that excluded 6 doctors (2 in the intervention group and 4 in the control group).	In the overall sample (Model 1), HPV vaccination coverage increased by 8.6 percentage points (intervention) and 6.4 percentage points (control). The treatment effect was not statistically significant according to a hierarchical linear model and an unstandardized coefficient (b) (b=0.023; SE=0.018; *p*<0.05). There was considerable variance in HPV vaccination coverage between physicians and clinics in Model 1, with the majority of the total variance lying with physicians (74%) vs. clinics (74%) vs. clinic level (14%).
Henrikson, 2015 ((20)	Mother’s score on the “Parental attitudes to childhood vaccines” test. Childhood vaccines by PACV percentage of mothers reluctant to vaccinate. Six single-item self-efficacy questions on communicating with parents about childhood vaccines (email survey).	Maternal vaccine hesitancy at 6 months (dichotomous). Maternal vaccine hesitancy at 6 months (ORDINAL measure).	The intervention had no effect on the mother’s vaccine hesitancy (*p*=0.78). Adjustment for baseline PACV score and race yielded similar results (OR: 1.22; 95% CI: 0.47–2.68; OR: 1 indicates no difference between the two groups).
Muñoz-Miralles, 2021 ((24)	Electronic medical records.	Vaccination rate.	The intervention was effective overall (OR: 2.48 [1.61–3.82]; *p*<0.001) and in people aged 60 and over (in good health, OR: 2.62 [1.32–5.17]; and with risk factors, OR: 2.95 [1.49–5.79]). There was no statistically significant difference in the efficacy of the intervention in people under 60 with risk factors, or between different diseases.
Szilagyi, 2021 ((21))	Electronic medical records.	Percentage of office visits with a missed HPV vaccination opportunity for vaccine initiation. Total number of missed opportunities for HPV vaccination. Proportion of adolescents receiving HPV vaccination.	The rate of missed opportunities decreased in intervention vs. control practices by 6.8% (95% CI: 3.9–9.7) for HPV vaccination initiation. No significant difference was observed for subsequent vaccination. The rate of missed opportunities decreased between the start of the study and the intervention period by 2.4% (95% CI: 1.2–3.5) in intervention vs. control practices. For adolescents with at least one office visit during the intervention period, HPV vaccine initiation was 3.4% (95% CI: 0.6–6.2) higher in intervention vs. control practices. No significant difference was observed for subsequent vaccination.

Abbreviations: aOR, adjusted odds ratio; CDC, Centers for Disease Control and Prevention; CI, confidence interval; COVID-19, coronavirus disease 2019; EMR, electronic medical records; HPV, human papillomavirus; PACV, Parental Attitudes on Childhood Vaccines score; OR, odds ratio; SE, standard error

### Effectiveness of communication training

#### Effective training

First, we note some training courses that proved effective in the HPV context. These included educational resources and patient-adapted recommendations. Following a self-guided webinar and two group sessions ((18)), the application of motivational interviewing during physician-patient interactions improved HPV vaccine adherence in adolescents. Similar training consisting of a webinar with three interactive modules and weekly encouragement to reveal common patient questions also improved vaccine adherence ((21)).

We also observed that good physician-patient communication includes a good understanding of the virus, the vaccine and the reasons for vaccine hesitancy. The study by Muñoz-Miralles *et al.* ((24)) shows a positive effect in patients aged 60 and over following a brief standardized intervention in the context of influenza. Although this communication depended on a directive guide, doctors and nurses were encouraged to adapt their communication by using empirical evidence to address the reasons for vaccine hesitancy, gathered beforehand.

This example can be enriched by the intervention proposed by Abdel-Qader *et al.* ((22)), who integrated the patient-partner perspective into the training material. The training, organized in 16 virtual sessions in a private Facebook group, invited pharmacists to be trained by eight doctors and eight pharmacists. However, the training sessions particularly included testimonials from patients discussing their experiences with the health crisis and vaccination. The patient-partner perspective justified the importance of patient-adapted communication. This study shows a significant reduction in vaccine hesitancy and an increase in vaccination rates. It should be noted, however, that the self-reported results of this study may be biased.

Training courses based on assertive communication cannot be overlooked. Brewer *et al.*’s study (17) demonstrated improved HPV vaccine adherence using an announcement, i.e., a vaccine recommendation given on the day of the consultation. The same study also evaluated the effectiveness of a conversation with the patient to present the vaccine for shared decision-making, but this intervention noted no significant difference.

### Risk of relative and cross-study bias

Seven studies ((16–19,21,23,24)) have low risk and two studies ((20,22)) are of some concern (see **Table 3**). A follow-up bias is present, as the healthcare professionals would have been aware of the result of randomizing to an intervention or control group. We consider this risk unavoidable, based on ethical considerations of informed consent, despite the fact that it may have had an impact on study results. The second bias ((20,22)) (measurement bias) is taken into account, since self-reported surveys were used, which can influence the validity of the results.

**Table 3:** Summary of risk of bias for included studies

Study - Cochrane RoB 2	Randomization bias	Follow-up bias	Attrition bias	Measurement bias	Evaluation and selection biases	Overall risk of bias
Abdel-Qader, 2022 ((22))	Low risk	Some concern	Low risk	Some concern	Low risk	Some concern
Boom, 2010 ((16))	Low risk	Some concern	Low risk	Low risk	Low risk	Low risk
Brewer, 2017 ((17))	Low risk	Low risk	Low risk	Low risk	Low risk	Low risk
Dempsey, 2018 ((18)	Low risk	Some concern	Low risk	Low risk	Low risk	Low risk
Gatwood, 2021 ((23)	Low risk	Some concern	Low risk	Low risk	Low risk	Low risk
Gilkey, 2019 ((19))	Low risk	Some concern	Low risk	Low risk	Low risk	Low risk
Henrikson, 2015 ((20)	Low risk	Some concern	Low risk	Some concern	Low risk	Some concern
Muñoz-Miralles, 2021 ((24)	Low risk	Low risk	Low risk	Low risk	Low risk	Low risk
Szilagyi, 2021 ((21))	Low risk	Low risk	Low risk	Low risk	Low risk	Low risk

Abbreviation: RoB 2, Risk of Bias Tool 2 for Randomized Controlled Trials

## Discussion

### Summary of levels of evidence

Randomized controlled trials evaluating the effectiveness of communication training for healthcare professionals are few in number and show mixed results in terms of vaccine adherence. Studies showing positive results have often adopted a communication approach aimed at formulating optimal recommendations and raising awareness of patients’ specific needs.

### Interpretations

The effectiveness of interventions does not seem to depend simply on the presence of communication that adopts epidemiological and medical knowledge, but also on communication that is adapted to the patient, understanding the factors that influence the vaccination decision. The most effective interventions ((24,25)) focused on HPV and targeted parents of minor patients. These studies have potentially been built on a better understanding of parental vaccine hesitancy, since the reasons for vaccine hesitancy and HPV have previously been addressed through research, improved communication and the development of quality recommendations ((25)). An adapted intervention, such as motivational interviewing ((18)), is consequently viewed favourably in the literature and by healthcare professionals ((6,26–28)). Infant vaccination (excluding HPV), on the other hand, seems to require more research, as indicated by studies by Brewer *et al*. and Henrikson *et al*. ((17,20)).

Contradictory results on the effectiveness of communication training can raise questions about the wider potential role of communication skills. In fact, communication in the therapeutic relationship is not limited exclusively to the transfer of medical knowledge about vaccination in clinical consultations. Both parties—the healthcare professional and the patient—are also influenced by societal communication, including socio-political and cultural factors that may be disseminated by public health authorities and popular rhetoric. In the case of HPV, linked to the sensitive subject of adolescent sexuality and gender ((29–31)), several socio-political factors have prompted a change in the public’s approach to vaccination ((32)). Social and medical perception seems to depend on multiple variables including ideology, customs, understanding of health, collective responsibility, trust and accessibility to healthcare ((33)).

Given the complexity of vaccine hesitancy, we would like to hypothesize that effective communication must take into account the above variables. The literature points to the inefficiency of a universal algorithm. In 2015, a systematic review on vaccine hesitancy demonstrated the need for a call for strategies tailored to the target population, the reasons for hesitancy and their context ((34)). We note in particular that effective studies tended to form recommendations with subjectivity according to the patient’s concerns, but the integration of all these variables remains to be applied to establish a bond of trust with patients. Further socio-culturally adapted communication interventions would be needed to study this topic.

## Limitations

There are several limitations to note. Other diversified studies would have enabled a better scope of conclusions, as well as a meta-analysis to understand the relationship between different groups of healthcare professionals, different diseases and vaccines, and then different communication training. Studies may be missing given the broad scope of the search strategy, the exclusion of articles published neither in English nor French, and the fact that only studies involving healthcare professionals authorized to administer the vaccine in Canada were included. Some studies also included different clinical locations and determining variables that may have been ignored or absent, such as regional infection rates, the context of the intervention (e.g. a national or regional vaccination program) and the demographics of specific patient groups. RCTs only were included in the study because of their rigorous methodology. It would also have been possible to include cohort studies with the same type of intervention.

## Conclusion

The effectiveness of vaccination-related communication varies according to the studies and knowledge acquired through training. This systematic review confirms the need for studies that focus on communication with healthcare professionals to build consensus around optimal, tailored training that increases trust in healthcare institutions. There is thus a need for studies that take into account initiatives that include the patient perspective in communication with healthcare professionals.

## Supplemental material

These documents are available in the Supplemental material file.
Supplemental material A: Search strategy
Supplemental material B: Effective communication strategies
Supplemental material C: Data extraction grid
